# T Cell Receptor Genotype and *Ubash3a* Determine Susceptibility to Rat Autoimmune Diabetes

**DOI:** 10.3390/genes12060852

**Published:** 2021-06-01

**Authors:** John P. Mordes, Laura Cort, Zhijun Liu, Ryan Eberwine, Elizabeth P. Blankenhorn, Brian G. Pierce

**Affiliations:** 1 Department of Medicine, University of Massachusetts Medical School, Worcester, MA 01655, USA; zhijun.liu@wfmc.edu.cn; 2 Department of Microbiology and Immunology, Center for Immunogenetics and Inflammatory Diseases, Drexel University College of Medicine, Philadelphia, PA 19129, USA; lc35@drexel.edu (L.C.); reberwine@me.com (R.E.); eb29@drexel.edu (E.P.B.); 3 Institute for Bioscience and Biotechnology Research, University of Maryland, Rockville, MD 20850, USA; pierce@umd.edu

**Keywords:** genetics, type 1 diabetes, immunogenetics, autoimmunity, MHC, TCR, rat

## Abstract

Genetic analyses of human type 1 diabetes (T1D) have yet to reveal a complete pathophysiologic mechanism. Inbred rats with a high-risk class II major histocompatibility complex (MHC) haplotype (RT1B/D^u^) can illuminate such mechanisms. Using T1D-susceptible LEW.1WR1 rats that express RT1B/D^u^ and a susceptible allele of the *Ubd* promoter, we demonstrate that germline knockout of *Tcrb-V13S1A1,* which encodes the Vβ13a T cell receptor β chain, completely prevents diabetes. Using the RT1B/D^u^-identical LEW.1W rat, which does not develop T1D despite also having the same *Tcrb-V13S1A1* β chain gene but a different allele at the *Ubd* locus, we show that knockout of the *Ubash3a* regulatory gene renders these resistant rats relatively susceptible to diabetes. In silico structural modeling of the susceptible allele of the Vβ13a TCR and its class II RT1^u^ ligand suggests a mechanism by which a germline TCR β chain gene could promote susceptibility to T1D in the absence of downstream immunoregulation like that provided by UBASH3A. Together these data demonstrate the critical contribution of the Vβ13a TCR to the autoimmune synapse in T1D and the regulation of the response by UBASH3A. These experiments dissect the mechanisms by which MHC class II heterodimers, TCR and regulatory element interact to induce autoimmunity.

## 1. Introduction

As with human type 1 diabetes (T1D), autoimmune diabetes occurs in various rat strains with a high-risk class II major histocompatibility complex (MHC) haplotype (*RT1.B/D^u^*) [1,2]. Among such rats, T1D is spontaneous in BBDP [3], Komeda [4] and LEW.1AR1 [5] rats, and it can be induced by immunological perturbation in LEW.1WR1 rats. More than 50 other loci, largely associated with immunoregulation, have been genetically mapped in humans and animals with T1D [6,7]. Among these are the genes encoding Ubiquitin-associated and SH3 domain-containing protein A (UBASH3A) [8,9] and diubiquitin (UBD) [10]. We have shown that *Ubd*-deficient LEW.1WR1 rats have reduced susceptibility to T1D [11].

A major rat diabetes gene is a T-cell receptor β chain variable (TCR-Vβ) region gene, *Tcrb-V13* [12]. A permissive allele for this gene, *Tcrb-V13S1A1*, which encodes Vβ13a, is expressed in six diabetes-susceptible, RT1.B/D^u^ rat strains (including LEW.1WR1), whereas three diabetes-resistant RT1.B/D^u^ strains express either *Tcrb-V13S1A2,* which encodes Vβ13b (e.g., WF rats), or carry the pseudo-gene *Tcrb-V13S1A3P* (F344 rats) [13,14]. We have shown that *Tcrb-V13S1A1* is a diabetes susceptibility gene by preventing diabetes with an allele-specific monoclonal antibody that depletes Vβ13a^+^ T-cells [15].

Here, using knockout technology, we prove the necessity of *Tcrb-V13S1A1* for susceptibility to T1D in the rat. We also determined that knockout of the T cell-expressed immunoregulatory gene *Ubash3a* unmasks T1D susceptibility in resistant LEW.1W rats. We then generated an in silico model of the high-risk immunologic synapse created by RT1B^u^ and Vβ13a. It revealed a plausible mechanism by which a germline TCR-Vβ gene and high-risk class II gene may confer susceptibility to T1D. These data illustrate the fundamental role of an autoimmune synapse that may (or may not) lead to overt disease depending on the downstream immunoregulatory environment.

## 2. Materials and Methods

### 2.1. Animals

Inbred diabetes-susceptible LEW.1WR1 and BBDP rats were obtained from BRM, Inc. (Worcester, MA, USA). They express the permissive RT1B/D^u^ class II MHC haplotype and the diabetes susceptibility allele of *Tcrb-V13S1A1*. LEW.1WR1 rats develop spontaneous diabetes at a rate of ~2.5%, but readily become diabetic after exposure to certain viruses and immunomodulators [3]. Nearly 100% of BBDP rats become spontaneously diabetic [3]. Inbred diabetes-resistant LEW.1W rats (that also carry RT1B/D^u^ and express *Tcrb-V13S1A1*) were obtained from a colony maintained in Worcester [11]. Animals were housed in viral antibody-free conditions, confirmed monthly to be serologically free of rat pathogens [12] and maintained according to institutional and national guidelines [16].

### 2.2. Diabetes Induction

To induce T1D, rats were injected with the viral-mimetic polyinosinic:polycytidylic acid (poly I:C) (1 μg/g body weight) three times weekly [17]. Rats were monitored for glycosuria, and diabetes was diagnosed when blood glucose concentration was >250 mg/dl on consecutive days using a handheld glucometer (One Touch). Animals were killed when diabetes was diagnosed or on day 40 after the first injection of poly I:C.

### 2.3. Vβ13a^+^ T Cell Depletion Studies

The 17D5 hybridoma produced the mouse anti-rat Vβ13 monoclonal antibody (mAb, IgG2a) that recognizes the “a” allotype of Vβ13 [14]. It depletes most Vβ13^+^ T cells and prevents diabetes in both LEW.1WR1 and BBDP rats [15]. Antibody was prepared as ascites, purified by affinity chromatography, and administered intraperitoneally (0.1 mg per rat in 0.5 mL). In studies using LEW.1WR1 rats, mAb was injected three times weekly. The first injection was given 48 h before the first injection of poly I:C. Spontaneously diabetic BBDP rats were injected with mAb once weekly beginning when diabetes was first detected at 45–60 days of age.

### 2.4. Generation of Vβ13 and Ubash3a Knockouts

Two pairs of zinc finger nucleases (ZFNs) targeting the coding region of the two genes of interest were designed by and purchased from Sigma Aldrich, ST. Louis MO.

For the Ubash3a knockout in the LEW.1W rat, the ZFN oligonucleotide is indicated in italics and the cut site is underlined: ATGCAGACGAACTAATGCTCAGTCCTGGTGAT*TACATCTTCGTGGACCCCACTCAGCAAGAAGAGGCCAGCGAG*GGCTGGGCCATTGGGATCTCACATC.

For the TCRVβ13 knockout in the LEW.1WR1 rat, we used this ZFN sequence indicated in italics and the cut site is underlined:

TTACAATCATTCCCATTTTGAAACACTATCTTCTTTTTCCACAGTTCTGTCTGAAACTGGAGTCACCCAGTCCCCCAGATATGCAATCATACAGGAAAGTCAGACTGTTTTCTTTTCGTGTGACCCTGTTTCTGGACATCAG*AGCCTTTACTGGTATCAGCAACACAGAGGCCAGGGGCCCCGGCT*TCTAGTTTACTTTCAGAATAAGGTTGATATAGATAAGTCACAGTTACCCAAGGATCGTTTTTTTGCTGTGAGGACTGAAGGAGTCAACTCCACTCTCAAGATCCAGTCTGCAAAGCAGAGTGACTCAGCCACC

Sigma provided quality control tests that indicated a cutting efficiency of ~12%. To prepare high quality DNA template for in vitro transcription, each of the two paired targeting ZFN-encoding expression plasmids was transformed into DH5a, and plasmid DNA was isolated using the GenElute HP endotoxin Free Plasmid Maxiprep kit (Sigma). The purified ZFN-encoding expression plasmids were linearized with XbaI (New England Biolabs), extracted with phenol/chloroform/isoamyl alcohol (Sigma) and precipitated with isopropanol (Fisher Scientific). Messenger RNA was in vitro transcribed, capped, and polyadenylated using the MessageMAX T7 ARCA-Capped Message Transcription Kit and Poly(A) polymerase Tailing Kit (Epicentre). The subsequent Poly(A) tailed ZFN mRNA was column purified with the MEGAclear kit (Ambion) before resuspension in RNAse free water. The mRNA was quantified using a NanoDrop-1000 (Fisher Scientific) and assessed for quality with an Agilent bioanalyzer (Agilent). The two paired ZFN mRNAs were combined in a concentration of 400 μg/mL in RNAse-free water. For pronuclear injection, the paired mRNAs were diluted to a final concentration of 10 ng/μL in 1mM Tris HCl, 0.1mM EDTA and kept on ice during the microinjection procedure. A Core Facility at UMass Medical School performed the injections of fertilized single cell embryos from superovulated rats, and the subsequent transfer to pseudopregnant females using standard procedures. One hundred fertilized 1 cell stage LEW.1WR1 or LEW.1W embryos were micro-injected with ZFN mRNA into the male pronucleus under standard conditions. The surviving embryos were implanted into pseudopregnant Sprague Dawley female rats and allowed to develop to term.

### 2.5. Genotyping

Genomic DNA was isolated from tail samples using GenElute Mammalian Genomic DNA miniprep kit (Sigma) and analyzed as described [13]. To identify mutants, gene-specific primers were used on genomic DNA to amplify the region by PCR. Primers used to detect the deletion from the ZFN were: UBASH3A Forward, CCTCCCACTGCTATTGCTTC and UBASH3A Reverse, AATGTGGCCAGTGTAGGGAC. For Vβ13, primers were: TCR-Vβ13 Forward: CACCAAGAGGCACTGTGCTA and TCR-Vβ13 Reverse, ATGGCCTGTGTCAAAAGACC.

### 2.6. Sequencing

Genes of interest were amplified from genomic DNA of diabetes-susceptible and -resistant rats and founder knockout rats using Hi-Fidelity Taq polymerase. The PCR products were purified and sequenced by Genewiz (South Plainfield, NJ, USA). The sequences were analyzed by 4peaks software (https://nucleobytes.com/4peaks/, http://nucleobytes.com/index.php/4peaks, accessed on 28 May 2021) and aligned by CLUSTALW (https://www.genome.jp/tools-bin/clustalw, accessed on 28 May 2021

### 2.7. Statistics

Statistical procedures were carried out using either GraphPad Prism (Ver. 6, La Jolla, CA, USA) or IBM SPSS Statistics (Armonk, NY, USA). Survival curves were analyzed using Kaplan-Meier methodology; equality of survival distributions was tested by log-rank statistic [18]. *p* values < 0.05 were considered statistically significant. Fisher’s exact statistic was used for analyzing 2 × 2 tables.

### 2.8. In Silico Modeling Procedures

We modeled the Vβ13a TCR and its recognition of insulin B:9-23 peptide bound to the rat class II MHC molecule, RT1B^u^, using previously described algorithms that predict structures of TCRs [19] and TCR-pMHC complexes [20]. We selected a rat Vα5-4 α chain as a partner to the Vβ13 TCR β chain, based on our finding that Vα5-4 RNA is expressed in cells within islets of pre-diabetic rats, and sequencing of multiple clones showed that it is focused [21]. The mouse homologue of this α chain has been associated with anti-insulin autoantibodies in the NOD mouse model of T1D [22]. The most common, focused (i.e., CDR3-identical) sequences of both Vβ13 and Vα5-4 were used; these were CAASSSYGANNKLTF (CDR3α) and CASSLGLGGETQYF (CDR3β). For the peptide autoantigen, we used insulin B:9-23 because studies have shown that insulin or this peptide is recognized by islet-infiltrating T-cells in mice and humans [23,24,25,26,27,28]. Furthermore, insulin B:9-23 (identical in rat, mouse and human) binds weakly to the diabetes-associated mouse class II molecule I-A^g7^ [22,29,30]. The TCR structure was modeled with TCRmodel (https://tcrmodel.ibbr.umd.edu, accessed on 28 May 2021), a template-based TCR modeling protocol [19]. The pMHC structure was generated with Modeller [31], with the MBP_125-135_/I-A^u^ structure (Protein Data Bank code 2P24) used as modeling template, based on MHC sequence identity (81%) of I-A^u^ and RT-1B^u^ (81%), as well as matching lengths of MHC helices for I-A^u^ and RT-1B^u^. The docking simulation to predict the TCR-pMHC interaction was performed using TCRFlexDock [20,32], with modeled TCR and pMHC structures as input. In accordance with the published protocol, TCRFlexDock was used to generate 1000 docking models by sampling rigid-body binding modes, as well as side chain and CDR loop conformations, during the docking search. All 1000 models were scored using the energetics-based interface score (I_sc) from Rosetta [33] to identify two candidate TCR-pMHC models, which correspond to models ranked #1 and #2 by I_sc.

## 3. Results

### 3.1. Production and Confirmation of Ubash3A and Tcrb-V13S1A1 Knockouts

**Figure 1:**
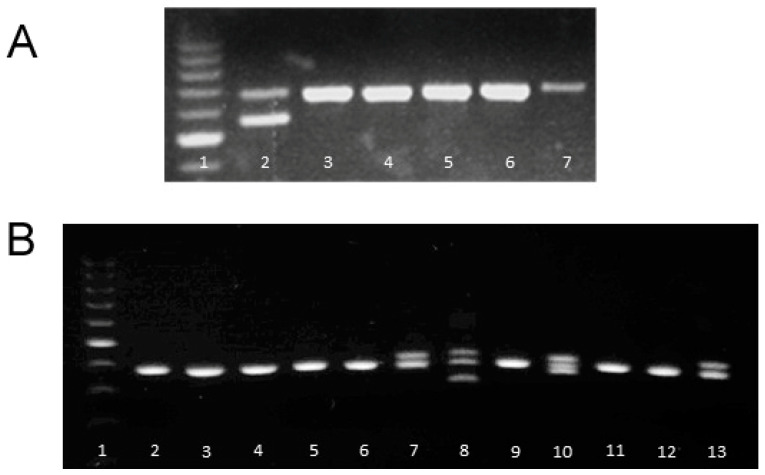
Panel (**A**). Heterozygous TCR-Vβ13 knockout founder among the first six LEW.1WR1 littermates (lane 2). The first lane is marker DNA; lanes 3–7 are wild-type. Panel (**B**). Heterozygous *Ubash3a* knockout founders among first derived progeny (lanes 7, 8, 10, and 13). Lane 1 is marker DNA; lanes 2–6, 9, 11–12 are samples from wild-type, non-deleted siblings. The founder used in this study is represented in lane 8 (Founder #7).

Knockout progeny were identified by PCR and gel electrophoresis using DNA from pups recovered from the first series of ZFN pronuclear injections (Figure 1A). One of them (Figure 1A, lane 2) had a deletion (113 bp). Sequencing the DNA from this founder showed that this excision results in a stop codon in exon 2 and functional deletion of this variable region. This heterozygous animal was used to create a homozygous knockout stock.

We used the same technology to knock out *Ubash3a* in T1D resistant LEW.1W rats to test the hypothesis that they would then become susceptible. LEW.1W rats differ from susceptible LEW.1WR1 rats with respect to the expression of diubiquitin (UBD) [11], but both share the same *Ubash3a* allele. Several LEW.1W-*Ubash3A* knockout heterozygotes (Figure 1B) were identified and one, Founder #7, was bred to a wild-type LEW.1W. Two heterozygous offspring were mated to produce homozygous gene knockout progeny. DNA from this line was sequenced, revealing that ZFN excision had deleted 67 base pairs in exon 6 (not shown), causing a frameshift.

### 3.2. Vβ13 Knockout LEW.1WR1 Rats Are Completely Diabetes Resistant

**Figure 2:**
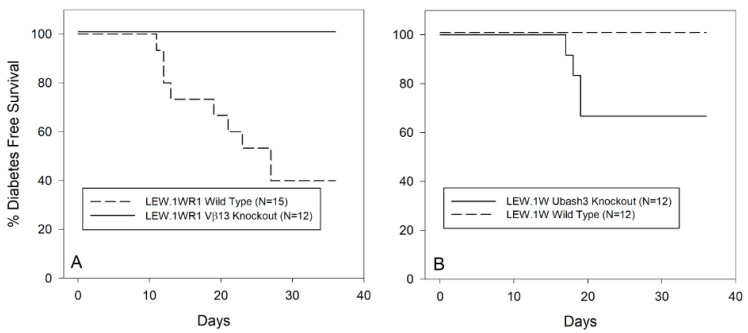
Panel (**A**). Frequency of diabetes in wild-type and *Vβ13S1A1^null/null^* LEW.1WR1 rats treated with poly I:C three times weekly as described in Methods. Rats deficient in Vβ13 are completely resistant to induction of T1D compared to parental strain, LEW.1WR1 rats. The two survival curves are statistically significantly different (*p* < 0.002). Panel (**B**). Frequency of diabetes in LEW.1W and LEW.1W *Ubash3a^null/null^* rats following treatment with poly I:C three times weekly as described in Methods. LEW.1W rats deficient in UBASH3A have significantly higher incidence of T1D than the resistant UBASH3A-sufficient LEW.1W parental strain (*p* < 0.04). Both strains carry *RT1.B/D^u^*.

Twelve Vβ13 homozygous knockout and 15 wild-type LEW.1WR1 rats of both sexes 21–25 days old were treated with poly I:C through 40 days of age. As shown in Figure 2A, 60% of wild-type animals became diabetic, a percentage consistent with previous reports of the susceptibility of this strain to perturbant-induced diabetes [17]. In contrast, none of 12 Vβ13 knockout rats became diabetic (*p* < 0.002).

### 3.3. Depletion of Vβ13a+ T Cells by Antibody Prevents T1D Only Early in Disease Progression

The Vβ13 knockout data suggested that this germline β chain is critical for T1D at the earliest stages of disease but left open the question of its necessity later in disease evolution. To address this question, we used the same depleting anti-Vβ13 mAb that is known to prevent disease when given early [15], to identify the time frame during which Vβ13+ T cells are necessary for disease.

As shown in Table 1, anti-Vβ13 mAb prevented T1D in LEW.1WR1 rats treated with poly I:C if started on day −5 or day +5 relative to the first dose of poly I:C but not if started on day +10, when insulitis is present but hyperglycemia is not (6/7 diabetic by day 40, *p* < 0.005).

We also investigated whether depletion of Vβ13^+^ T cells immediately after onset of diabetes could reverse the disease. We first tested BBDP rats that had become spontaneously diabetic between 45–60 days of age. Continuous weekly anti-Vβ13 mAb given for one month beginning on the day of diabetes detection failed to reverse the disease (N = 4).

Similarly, weekly anti-Vβ13 mAb given for one month beginning on the day of diabetes detection did not normalize glycemia in wild-type LEW.1WR1 rats that had been treated with poly I:C (N = 7). During the study, diabetic animals were treated with small daily injections of regular human insulin that were sufficient to prevent ketoacidosis but not normalize glucose.

### 3.4. Ubash3A Knockout LEW.1W Rats Become Diabetes Susceptible

We compared susceptibility to poly I:C-induced diabetes in LEW.1W *Ubash3a*-knockout rats (N = 12) with wild-type LEW.1W rats (N = 12). As shown in Figure 2B, no wild-type LEW.1W animals became diabetic, whereas 5 of 12 knockout rats became diabetic (*p* < 0.04). In comparison to T1D-susceptible LEW.1WR1 rats treated in the same manner (Figure 2A), the *Ubash3a* knockout rats had intermediate susceptibility.

### 3.5. In Silico Modeling of a Vβ13 TCR and the Mechanistic Basis of Its Autoimmune Recognition

From these and previously reported results, it is likely that the genes encoding UBD, UBASH3A and TCRVβ13a participate in pathways that initiate and lead to diabetes. The mechanism of diabetes resistance attributable to UBASH3A is probably related to its role in suppressing T cell activity [9]. The role of *Ubd* in T1D may be due to its influence on *Cblb*, a key modulator of T cell activation [11] and a major susceptibility gene in Komeda rats [4].

In contrast, the singular importance of one specific TCR variable region is more puzzling. The allelic differences between Vβ13a and Vβ13b that predict T1D susceptibility or resistance, respectively, are restricted to the CDR1 and CDR2 regions, where three amino acid changes occur (Q30E and S31R in CDR1; K59E in CDR2 [14]).

#### 3.5.1. TCR and Insulin B:9-23 Peptide-MHC Structures

Knowing that all diabetes-susceptible rat strains carry the RT1B/D^u^ MHC haplotype and the *Vβ13S1a* allele [14,15], we explored the geometry of the predicted diabetes-related TCR-peptide-MHC complex using in silico modeling. We used a Vβ13a TCR β chain and Vα5 TCR α chain sequences that were abundant in T cells cloned from cell cultures of diabetic LEW.1WR1 islets [21]. The TCR structure was modeled using TCRmodel, a TCR structure modeling web server that integrates template-based TCR modeling with energy minimization, and is implemented in the Rosetta software package [19]. We used the Modeller program [31] to generate a homology model of the high-risk class II RT1-B^u^ molecule for MHC in complex with the insulin B:9-23 peptide.

**Figure 3:**
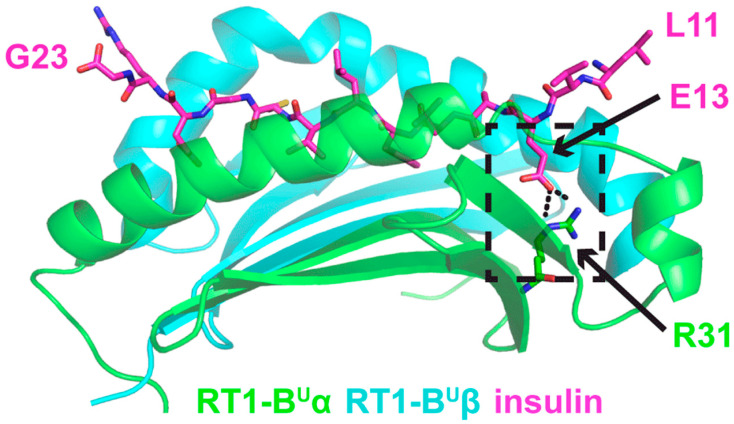
Structural model of insulin B peptide presented by RT1-B^u^ MHC. RT1-B^u^ α and β chains are shown as cartoons and colored green and cyan, respectively. Insulin B residues 11-23 are present in the model, and are shown in stick representation, colored magenta. Putative salt bridge interaction between peptide residue glutamic acid 13 (E13) and MHC α residue arginine 31 (R31) is shown in the black dashed box.

The model is shown in Figure 3. Insulin peptide residues 9 and 10 are not present in the coordinates of the structural model, as corresponding peptide residues are not present in the structural template, and they are predicted to be outside the MHC binding groove and distal from the TCR interaction site, thus likely irrelevant for complex formation. This peptide register on the MHC is reflective of the previously reported X-ray structure of this same peptide to the human DQ8 MHC [23], where insulin peptide residues 9 and 10 are likewise not present. This complex is predicted to be stabilized by a salt bridge interaction between insulin-E13 (glutamic acid at amino acid 13 of the insulin peptide) and arginine R31 of the MHC α chain in the RT1-B^u^ P1 pocket (Figure 3). Of great interest, in RT1-B^L^, residue 31 is glutamic acid [34], which would be unfavorable for interaction with the glutamic acid at insulin residue E13 due to electrostatic repulsion, and thus, may explain why RT1-B^L^ is a resistant MHC haplotype.

#### 3.5.2. Model 1

**Figure 4:**
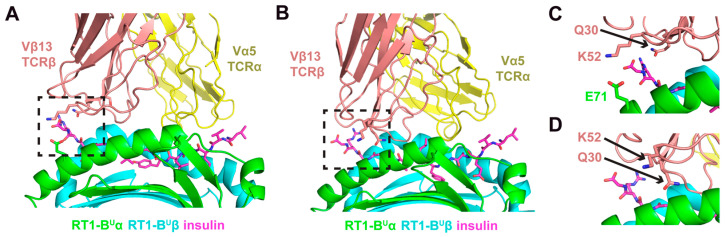
Two models of the putative recognition between the TCR and insulin peptide-MHC. (**A**) Model A shows the TCR with a canonical diagonal binding mode over the peptide, with a calculated crossing angle of 29° and limited tilt (4.8°), and the Vβ13 domain positioned over the C-terminal half of the peptide, which, as in Figure 3, is on the left. (**B**) Model B exhibits an alternative TCR engagement mode of peptide-MHC. For that model, a more tilted docking orientation (calculated tilt angle 16.5°) that leads to a greater engagement of the peptide MHC by the Vβ chain relative to the Vα chain. (**C**) A close-up view of the interface in Model A (boxed region in panel (**A**)) shows two Vβ13 CDR1 and CDR2 loop residues with identities specific to the Vβ13a allele (Q30 and K52) that are positioned to readily interact with the peptide-MHC. (**D**) A close-up view of the interface in Model B (boxed region in panel (**B**)) indicates that residue Q30 is likely to interact with the peptide-MHC, whereas K52 is not predicted to contact the peptide or MHC in that model. In all panels, peptide residues are shown in magenta with stick representation, and Vβ13 residues Q30 and K52 are shown in stick representation. MHC α chain residue E71, which is proximal to TCRβ residue Κ52, is shown in stick representation in panels (**A**) and (**C**). Close-up views in (**C**) and (**D**) are rotated slightly with respect to panels (**A**) and (**B**), to more clearly distinguish key residues.

With the modeled TCR and insulin B:9-23 peptide-MHC structures, we generated two models of the putative recognition between the TCR and peptide-MHC using a previously described TCR docking algorithm [20] (Figure 4). The first model (Model A) shows the TCR with a canonical diagonal binding mode over the peptide [35], with a calculated crossing angle [35] of 29°, and the Vβ13 domain positioned over the C-terminal half of the peptide. A more detailed inspection of this interface shows that, in addition to CDR3 contacts (not highlighted in this representation), two Vβ13 CDR1 and CDR2 loop residues with identities specific to the Vβ13a allele [14] are positioned to readily interact with the peptide-MHC (Figure 4C). Specifically, residue glutamine 30 (Q30) in the CDR1 loop is proximal (4.6 Å) to the insulin peptide, while residue lysine 52 (K52) in the CDR2 loop is proximal (4.5 Å) to glutamic acid 71 (E71) on the RT-1B^u^ MHC α chain, indicating a possible salt bridge interaction. Notably, the E30 and the S52 residues in diabetes-resistant Vβ13b would change the charge of these interactions. Importantly, these are among the residues that are different between Vβ13a and Vβ13b.

#### 3.5.3. Model 2

A second model generated by our docking simulations, which also exhibited a highly favorable energetic score, is shown in Figure 4B, and exhibits an alternative TCR engagement mode with peptide-MHC. For that model, Model B, a more tilted docking orientation leads to a greater engagement of the peptide-MHC by the Vβ chain relative to the Vα chain. The tilt or incident angle [20] for this model was calculated to be 16.5°, much greater than the tilt angle of Model A (4.8°); and is greater than that of 83% of 54 experimentally determined Class II TCR-pMHC complex structures from the TCR3d database [36]. Such tilted orientations have been associated with autoimmune TCR recognition [37], and include recently reported structures of murine TCRs in complex with insulin and IA^g7^ Class II MHC (Protein Data Bank codes 6DFS, 6DFW) [38]; these have tilt angles of 16.7° and 17.3° reported in TCR3d, which are highly similar to the tilt of Model B. Inspection of residues Q30 and K52, which are specific to the Vβ13a allele, in Model B (Figure 4D) indicates that residue Q30 is likely to interact with the peptide-MHC, whereas K52 is not predicted to contact the peptide or MHC in that model. As noted above, these are critical elements that distinguish Vβ13a from Vβ13b and suggest a mechanism underlying their differential association with T1D susceptibility and resistance, respectively.

## 4. Discussion

We report here data that contribute to our understanding of the interactive roles of three genes in susceptibility to T1D-like autoimmune disease in the rat. These studies prove the essential role of Tcrb-V13a in disease susceptibility and suggest by in silico analysis a mechanism by which this can occur in the context of the required class II allotype. Conversely, the genetic loss of a single immunomodulatory gene, *Ubash3a*, renders resistant rats (which have the necessary TCR and MHC but lack a high level of UBD expression) susceptible to T1D.

*Tcrbv13* plays a critical role in genetic susceptibility to rat T1D. The evidence was initially based on gene mapping [39] and then functional studies showing that depletion of Vβ13a^+^ T cells prevents spontaneous and poly I:C-induced [15] autoimmune diabetes. The importance of *Tcrbv13* in the earliest stage of diabetes pathogenesis is now confirmed by demonstrating that genetic deletion of this TCR β chain completely prevents disease whereas removal of Vβ13+ T cells cannot prevent incipient or established diabetes. In their aggregate, these observations strongly suggest, but do not prove, that the TCR encoded by the susceptible allele of *Tcrbv13,* Vβ13a, is critical at the very beginning of the diabetogenic process, probably in the thymus, when it encounters autoantigenic peptide in the context of RT1B/D^u^ MHC.

The importance of genomically encoded elements in the TCR is supported by our previous studies demonstrating that Vβ13a is not dependent on any one V(D)J recombination event. We showed that CDR3 sequences were not predictive, as long as they are permissive [21]. Together with the requirement for MHC class II RT1B/D^u^ in rat T1D, the data clearly imply the existence of a “diabetogenic immunological synapse” between MHC and genomically encoded TCR sequences that predispose to disease. The data also explain the resistance of RT1B/D^u^ WF rats because they express the resistant *TcrbV-13S1A2* allele [12,13].

The availability of the LEW.1W rat, which does not develop T1D in response to poly I:C despite expressing RT1B/Du and Vβ13a, has allowed us to explore “downstream” events, specifically the roles of both protective and pro-diabetic candidate genes. We have previously shown that *Ubd* facilitates diabetes expression in the LEW.1WR1 rat and that its expression is reduced in the resistant LEW.1W rat [11]. This favorable genetic constellation enabled us to demonstrate here that a small, targeted disturbance in the immunoregulatory environment caused by genetic deletion of *Ubash3a* also affects disease expression. The genetic association between a SNP in the sixth intron of UBASH3A and human T1D has been reported [40]. UBASH3A is also called suppressor of T-cell signaling (STS-2), T-cell ubiquitin ligand (TULA), and Cbl-interacting protein 4 (CLIP4), and is expressed predominately in T and B lymphocytes where it acts as a negative regulator of lymphocyte activation [41]. This and the fact that *Cblb* is a documented susceptibility gene in diabetic Komeda rats [4] makes *Ubash3a* a plausible candidate for a T1D susceptibility gene. Although the deletion of *Ubash3a* in rats resulted in enhanced diabetes induction in the LEW.1W knockout, the incidence of T1D did not achieve the degree of penetrance of that seen in the fully susceptible LEW.1WR1, from which it also differs at the *Ubd* locus. This is consistent with research on similar mouse knockout lines carrying deletions of *Ubash3a* and/or *Ubash3b*, that the two related genes are functionally synergistic [9,42] at the level of T cell suppression.

The in silico studies reported here were performed to explore the fundamental mechanism underlying the genetic susceptibility conferred by *Tcrbv-13a*. Analysis of computational docking models support likely positioning of Vβ CDR1 and CDR2 loops in contact with the target peptide and MHC. These are the regions that distinguish the diabetogenic and resistant alleles of Vβ13, and modeled structures indicate that the allele-specific residues can readily interact with the peptide and/or MHC. These observations are consistent with previous reports of the importance of these regions in the immunological synapse (reviewed in [43]). Both regions are critical for T cell-MHC restriction [44], and it has been reported that they interact with MHC helices to produce unanticipated and potentially important effects. Biased TCR usage against HLA DQ8-restricted gliadin peptides has been observed in human celiac disease patients [45], where TCR usage biased to TRBV9*01 underpins the recognition of HLA-DQ8-α-1-gliadin. More importantly for our hypothesis, these authors observed that, “…all CDRβ loops [not just CDR3] interact with the gliadin peptide” and that “…Leu37β from the CDR1β loop, and Tyr57β from the CDR2β loop are the ‘hot spot’ residues underpinning the TCR-DQ8-gliadin-α1 interaction providing a basis for the TRBV9*01 bias” in celiac disease. Another crystallographic study was designed specifically to address the question of whether shared germline contacts within the TCR-pMHC would persist despite distinct CDR3-peptide contacts in the model system—and they do [46]. The authors concluded that “…a TCR utilizing entirely distinct chemistries to recognize different peptides exhibits highly persistent germline-mediated contacts.”

Model B is possibly more intriguing, showing that the rat T1D-InsB9:23-Vβ13a/Vα5 TCR may adopt a “tilted” binding orientation. Similar tilted binding orientations have been observed for other autoimmune-associated TCRs complexes, and are thought to enable autoimmune TCRs to bypass thymic deletion [37]. For example, two human TCRs from multiple sclerosis patients (Hy.1B11 and Ob.1A12) both engage a myelin basic protein (MBP) peptide and an MHC molecule with tilted geometries [47,48]. Other examples of experimentally determined structures with tilted TCR engagement of Class II MHCs include two TCRs that interact with insulin B and IA^g7^ [38], two human TCRs that interact with a mutant form of human triosephosphate isomerase peptide presented by HLA-DR1 [49,50], and a human TCR (S2) that interacts with the HLA-DQ2-gliadin antigen in celiac disease [51]. Though Models A and B await experimental confirmation, they provide encouraging support for our hypothesis. Experimental structural determination, or model-guided mutagenesis and binding measurements to assess role of putative TCR interface residues, can be used in prospective studies to further define the mechanistic role of Vβ13a residues.

We recognize that our analysis of the TCR-pMHC complex incorporates several assumptions: a putative α chain, a putative peptide, and one of several permissive CDR3 sequences. However, although there is no one obligate α chain and no preferred CDR3 sequence [21], our selections are reasonable. Similarly, insulin B:9-23 may not be the only autoantigenic peptide, but an extensive literature suggests that it is a likely one [27]. What is important is that the model we created using these choices also used MHC and TCR β chains that are conclusively known to be a part of the diabetogenic process, showing that canonical and tilted TCR docking orientations (Models A and B, respectively) enable key allele-specific residues to form contacts with the peptide-MHC.

## 5. Conclusions

These results provide unique insight into the pathogenesis of T1D because, in the rat, two obligate susceptibility genes (*Vβ13* and *RT1B/Du*), one strong resistance gene (*Ubash3a*), and one penetrance-enhancing gene (*Ubd*) have been identified and incorporated into a plausible, granular disease model, something not yet achieved in studies of human T1D. Variants of other resistance genes are likely act stoichiometrically to influence disease progression. In summary, elimination of Vβ13a^+^ cells in young rats or inheritance of a resistance allele at *Tcrvb13* prevents disease. The other genes we have identified are downstream modulators that facilitate or inhibit the process that started earlier in the immune synapse. These data from rat models clarify the immunologic landscape and underlying mechanisms of T1D as they apply to different members of a species to different degrees. If it were to be found that specific pairs of MHC and TCR confer susceptibility to human T1D, then a highly targeted TCR deletional strategy could arrest disease development.

## Figures and Tables

**Table 1:** Diabetes in LEW.1WR1 Rats.

Start of Anti-Vβ13 mAb Relative to Poly I:C	Day −5 or +5	Day +10
Diabetic	1	6
Non-diabetic	9	1

## Data Availability

**I Data and Resource Availability:** LEW.1W and LEW.1WR1 rats are available commercially. LEW.1WR1-*TcrVβ13* knockout rats are available by application to Dr. Liu. The LEW.1W-*Ubash3a* knockout rats are no longer available.

## References

[1] Easterfield A.J., Bradley J.A., Bolton E.M. (2003). Complementary DNA sequences encoding the rat MHC class II RT1-Bu and RT1-Du alpha and beta chains. Immunogenetics.

[2] Ellerman K.E., Like A.A. (2000). Susceptibility to diabetes is widely distributed in normal class IIu haplotype rats. Diabetologia.

[3] Mordes J.P., Bortell R., Blankenhorn E.P., Rossini A.A., Greiner D.L. (2004). Rat models of type 1 diabetes: Genetics, environment, and autoimmunity. Ilar J. Natl. Res. Counc. Inst. Lab. Anim. Resour..

[4] Yokoi N., Hayashi C., Fujiwara Y., Wang H.Y., Seino S. (2007). Genetic reconstitution of autoimmune type 1 diabetes with two major susceptibility genes in the rat. Diabetes.

[5] Jörns A., Akin M., Arndt T., Terbish T., Zu Vilsendorf A.M., Wedekind D., Hedrich H.J., Lenzen S. (2014). Anti-TCR therapy combined with fingolimod for reversal of diabetic hyperglycemia by β cell regeneration in the LEW.1AR1-iddm rat model of type 1 diabetes. J. Mol. Med..

[6] Wang Z., Xie Z., Lu Q., Chang C., Zhou Z. (2017). Beyond Genetics: What Causes Type 1 Diabetes. Clin. Rev. Allergy Immunol..

[7] Bradfield J.P., Qu H.Q., Wang K., Zhang H., Sleiman P.M., Kim C.E., Mentch F.D., Qiu H., Glessner J.T., Thomas K.A. (2011). A genome-wide meta-analysis of six type 1 diabetes cohorts identifies multiple associated loci. PLoS Genet..

[8] Todd J.A. (2018). Evidence that UBASH3 is a causal gene for type 1 diabetes. Eur. J. Hum. Genet..

[9] Ge Y., Paisie T.K., Newman J.R.B., McIntyre L.M., Concannon P. (2017). UBASH3A Mediates Risk for Type 1 Diabetes Through Inhibition of T-Cell Receptor-Induced NF-κB Signaling. Diabetes.

[10] Aly T.A., Baschal E.E., Jahromi M.M., Fernando M.S., Babu S.R., Fingerlin T.E., Kretowski A., Erlich H.A., Fain P.R., Rewers M.J. (2008). Analysis of single nucleotide polymorphisms identifies major type 1A diabetes locus telomeric of the major histocompatibility complex. Diabetes.

[11] Cort L., Habib M., Eberwine R.A., Hessner M.J., Mordes J.P., Blankenhorn E.P. (2014). Diubiquitin (Ubd) is a susceptibility gene for virus-triggered autoimmune diabetes in rats. Genes Immun..

[12] Mordes J.P., Leif J., Novak S., DeScipio C., Greiner D.L., Blankenhorn E.P. (2002). The iddm4 locus segregates with diabetes susceptibility in congenic WF.iddm4 rats. Diabetes.

[13] Mordes J.P., Cort L., Norowski E., Leif J., Fuller J.M., Lernmark A., Greiner D.L., Blankenhorn E.P. (2009). Analysis of the rat Iddm14 diabetes susceptibility locus in multiple rat strains: Identification of a susceptibility haplotype in the Tcrb-V locus. Mamm. Genome.

[14] Stienekemeier M., Hofmann K., Gold R., Herrmann T. (2000). A polymorphism of the rat T-cell receptor beta-chain variable gene 13 (BV13S1) correlates with the frequency of BV13S1-positive CD4 cells. Immunogenetics.

[15] Liu Z., Cort L., Eberwine R., Herrmann T., Leif J.H., Greiner D.L., Yahalom B., Blankenhorn E.P., Mordes J.P. (2012). Prevention of type 1 diabetes in the rat with an allele-specific anti-T-cell receptor antibody: Vbeta13 as a therapeutic target and biomarker. Diabetes.

[16] Institute of Laboratory Animal Research, Committee on Care, & Use of Laboratory Animals, National Research Council (1996). Guide for the Care and Use of Laboratory Animals.

[17] Mordes J.P., Guberski D.L., Leif J.H., Woda B.A., Flanagan J.F., Greiner D.L., Kislauskis E.H., Tirabassi R.S. (2005). LEW.1WR1 rats develop autoimmune diabetes spontaneously and in response to environmental perturbation. Diabetes.

[18] Bewick V., Cheek L., Ball J. (2004). Statistics review 12: Survival analysis. Crit. Care.

[19] Gowthaman R., Pierce B.G. (2018). TCRmodel: High resolution modeling of T cell receptors from sequence. Nucleic Acids Res..

[20] Pierce B.G., Weng Z. (2013). A flexible docking approach for prediction of T cell receptor-peptide-MHC complexes. Protein Sci..

[21] Eberwine R.A., Cort L., Habib M., Mordes J.P., Blankenhorn E.P. (2014). Autoantigen-induced focusing of Vβ13+ T cells precedes onset of autoimmune diabetes in the LEW.1WR1 rat. Diabetes.

[22] Nakayama M., Castoe T., Sosinowski T., He X., Johnson K., Haskins K., Vignali D.A., Gapin L., Pollock D., Eisenbarth G.S. (2012). Germline TRAV5D-4 T-cell receptor sequence targets a primary insulin peptide of NOD mice. Diabetes.

[23] Lee K.H., Wucherpfennig K.W., Wiley D.C. (2001). Structure of a human insulin peptide-HLA-DQ8 complex and susceptibility to type 1 diabetes. Nat. Immunol..

[24] Pugliese A. (2017). Autoreactive T cells in type 1 diabetes. J. Clin. Investig..

[25] Zhang L., Jasinski J.M., Kobayashi M., Davenport B., Johnson K., Davidson H., Nakayama M., Haskins K., Eisenbarth G.S. (2009). Analysis of T cell receptor beta chains that combine with dominant conserved TRAV5D-4*04 anti-insulin B:9-23 alpha chains. J. Autoimmun..

[26] Latek R.R., Suri A., Petzold S.J., Nelson C.A., Kanagawa O., Unanue E.R., Fremont D.H. (2000). Structural basis of peptide binding and presentation by the type I diabetes-associated MHC class II molecule of NOD mice. Immunity.

[27] Sosinowski T., Eisenbarth G.S. (2013). Type 1 diabetes: Primary antigen/peptide/register/trimolecular complex. Immunol. Res..

[28] Landry L.G., Anderson A.M., Russ H.A., Yu L., Kent S.C., Atkinson M.A., Mathews C.E., Michels A.W., Nakayama M. (2021). Proinsulin-Reactive CD4 T Cells in the Islets of Type 1 Diabetes Organ Donors. Front. Endocrinol..

[29] Levisetti M.G., Suri A., Petzold S.J., Unanue E.R. (2007). The insulin-specific T cells of nonobese diabetic mice recognize a weak MHC-binding segment in more than one form. J. Immunol..

[30] Stadinski B.D., Zhang L., Crawford F., Marrack P., Eisenbarth G.S., Kappler J.W. (2010). Diabetogenic T cells recognize insulin bound to IAg7 in an unexpected, weakly binding register. Proc. Natl. Acad. Sci.USA.

[31] Eswar N., Webb B., Marti-Renom M.A., Madhusudhan M.S., Eramian D., Shen M.Y., Pieper U., Sali A. (2006). Comparative protein structure modeling using Modeller. Curr. Protoc. Bioinform..

[32] Pierce B., Weng Z. (2008). A combination of rescoring and refinement significantly improves protein docking performance. Proteins.

[33] Leaver-Fay A., Tyka M., Lewis S.M., Lange O.F., Thompson J., Jacak R., Kaufman K., Renfrew P.D., Smith C.A., Sheffler W. (2011). ROSETTA3: An object-oriented software suite for the simulation and design of macromolecules. Methods Enzym..

[34] Chao N.J., Timmerman L., McDevitt H.O., Jacob C.O. (1989). Molecular characterization of MHC class II antigens (beta 1 domain) in the BB diabetes-prone and -resistant rat. Immunogenetics.

[35] Rudolph M.G., Stanfield R.L., Wilson I.A. (2006). How TCRs bind MHCs, peptides, and coreceptors. Annu. Rev. Immunol..

[36] Gowthaman R., Pierce B.G. (2019). TCR3d: The T cell receptor structural repertoire database. Bioinformatics.

[37] Yin Y., Li Y., Mariuzza R.A. (2012). Structural basis for self-recognition by autoimmune T-cell receptors. Immunol. Rev..

[38] Wang Y., Sosinowski T., Novikov A., Crawford F., White J., Jin N., Liu Z., Zou J., Neau D., Davidson H.W. (2019). How C-terminal additions to insulin B-chain fragments create superagonists for T cells in mouse and human type 1 diabetes. Sci. Immunol..

[39] Martin A.M., Blankenhorn E.P., Maxson M.N., Zhao M., Leif J., Mordes J.P., Greiner D.L. (1999). Non-major histocompatibility complex-linked diabetes susceptibility loci on chromosomes 4 and 13 in a backcross of the DP-BB/Wor rat to the WF rat. Diabetes.

[40] Concannon P., Onengut-Gumuscu S., Todd J.A., Smyth D.J., Pociot F., Bergholdt R., Akolkar B., Erlich H.A., Hilner J.E., Julier C. (2008). Type 1 Diabetes Genetics, C., A human type 1 diabetes susceptibility locus maps to chromosome 21q22.3. Diabetes.

[41] Tsygankov A.Y. (2013). TULA-family proteins: A new class of cellular regulators. J. Cell Physiol..

[42] San Luis B., Sondgeroth B., Nassar N., Carpino N. (2011). Sts-2 is a phosphatase that negatively regulates zeta-associated protein (ZAP)-70 and T cell receptor signaling pathways. J. Biol. Chem..

[43] Wucherpfennig K.W. (2010). The first structures of T cell receptors bound to peptide-MHC. J. Immunol..

[44] Sim B.C., Zerva L., Greene M.I., Gascoigne N.R. (1996). Control of MHC restriction by TCR Valpha CDR1 and CDR2. Science.

[45] Broughton S.E., Petersen J., Theodossis A., Scally S.W., Loh K.L., Thompson A., van Bergen J., Kooy-Winkelaar Y., Henderson K.N., Beddoe T. (2012). Biased T cell receptor usage directed against human leukocyte antigen DQ8-restricted gliadin peptides is associated with celiac disease. Immunity.

[46] Adams J.J., Narayanan S., Liu B., Birnbaum M.E., Kruse A.C., Bowerman N.A., Chen W., Levin A.M., Connolly J.M., Zhu C. (2011). T cell receptor signaling is limited by docking geometry to peptide-major histocompatibility complex. Immunity.

[47] Sethi D.K., Gordo S., Schubert D.A., Wucherpfennig K.W. (2013). Crossreactivity of a human autoimmune TCR is dominated by a single TCR loop. Nat. Commun..

[48] Hahn M., Nicholson M.J., Pyrdol J., Wucherpfennig K.W. (2005). Unconventional topology of self peptide-major histocompatibility complex binding by a human autoimmune T cell receptor. Nat. Immunol..

[49] Deng L., Langley R.J., Brown P.H., Xu G., Teng L., Wang Q., Gonzales M.I., Callender G.G., Nishimura M.I., Topalian S.L. (2007). Structural basis for the recognition of mutant self by a tumor-specific, MHC class II-restricted T cell receptor. Nat. Immunol..

[50] Deng L., Langley R.J., Wang Q., Topalian S.L., Mariuzza R.A. (2012). Structural insights into the editing of germ-line-encoded interactions between T-cell receptor and MHC class II by Valpha CDR3. Proc. Natl. Acad. Sci. USA.

[51] Petersen J., Montserrat V., Mujico J.R., Loh K.L., Beringer D.X., van Lummel M., Thompson A., Mearin M.L., Schweizer J., Kooy-Winkelaar Y. (2014). T-cell receptor recognition of HLA-DQ2-gliadin complexes associated with celiac disease. Nat. Struct. Mol. Biol..

